# Supplementary figures and images for: Comparative analysis of the unbinding pathways of antiviral drug Indinavir from HIV and HTLV1 proteases by supervised molecular dynamics simulation
Source: PLoS One. 2021 Sep 27;16(9):e0257916. doi: 10.1371/journal.pone.0257916 (PMC8476009; doi:10.1371/journal.pone.0257916)

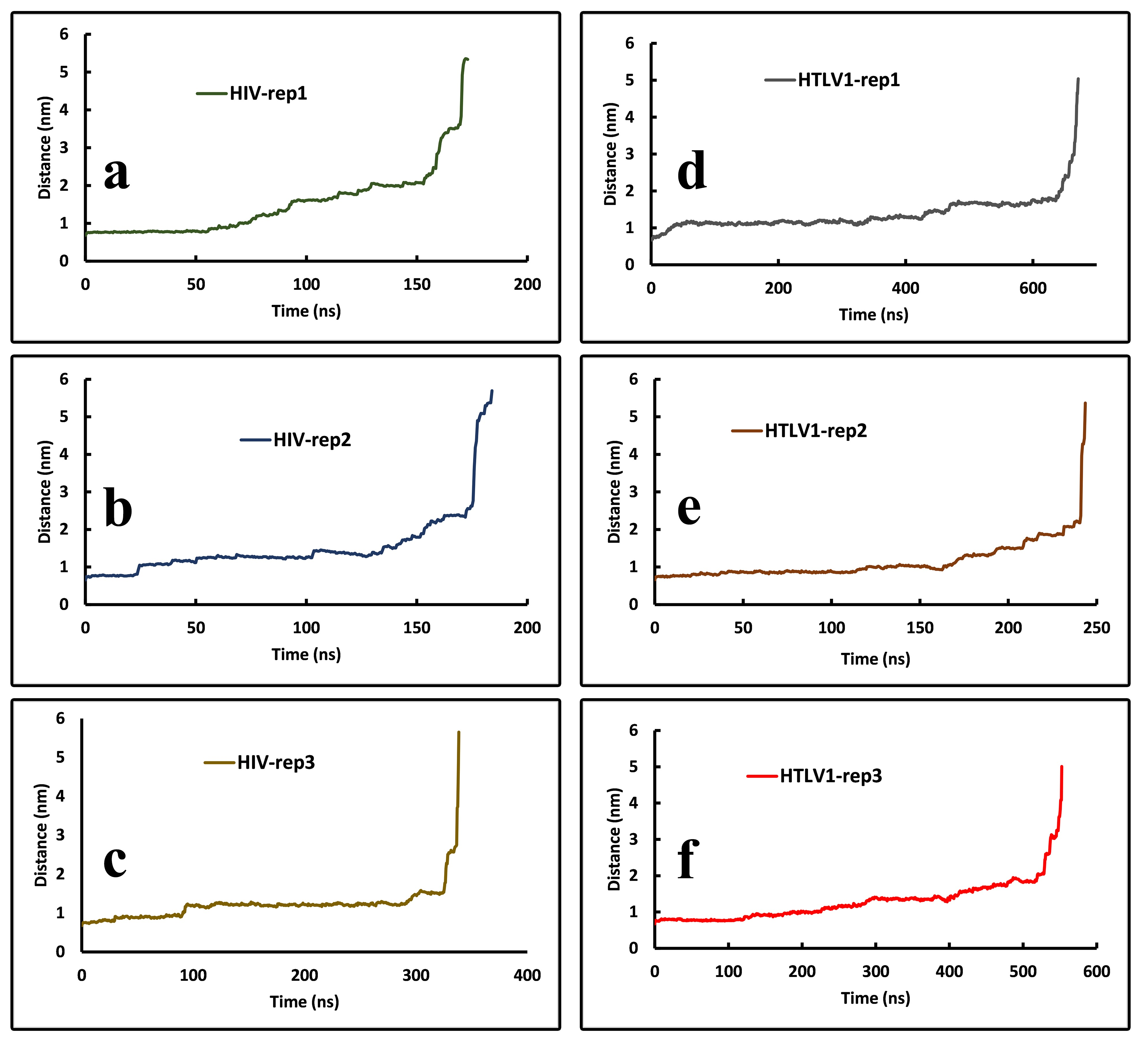

Supplement: S1 Fig — a, b, c, The HIV protease, d, e, f, The HTLV-1 protease. (TIF) [file pone.0257916.s001.tif]

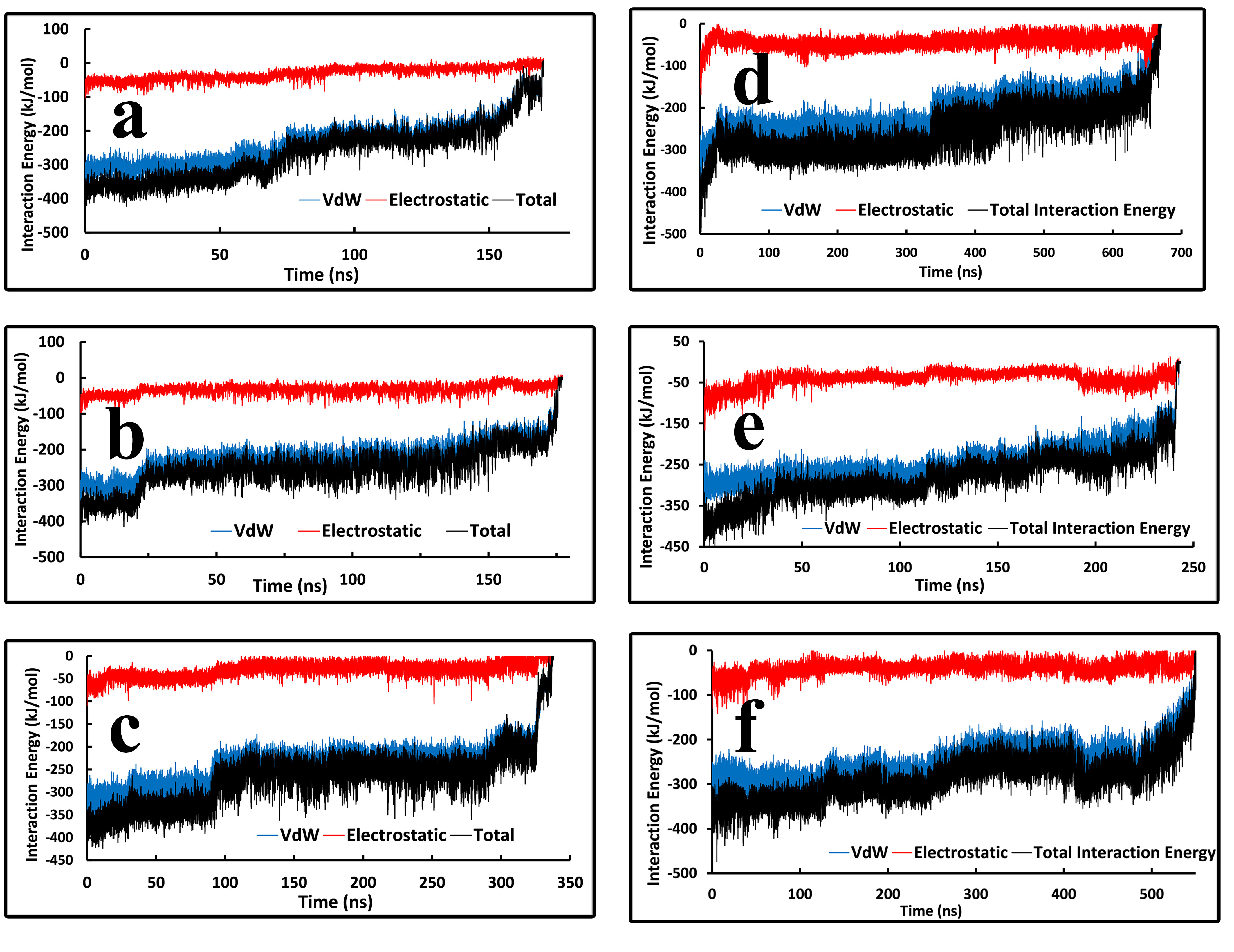

Supplement: S2 Fig — a, b, c, The Indinavir-HIV complex and, d, e, f, The Indinavir- HTLV-1 protease complex. (TIF) [file pone.0257916.s002.tif]

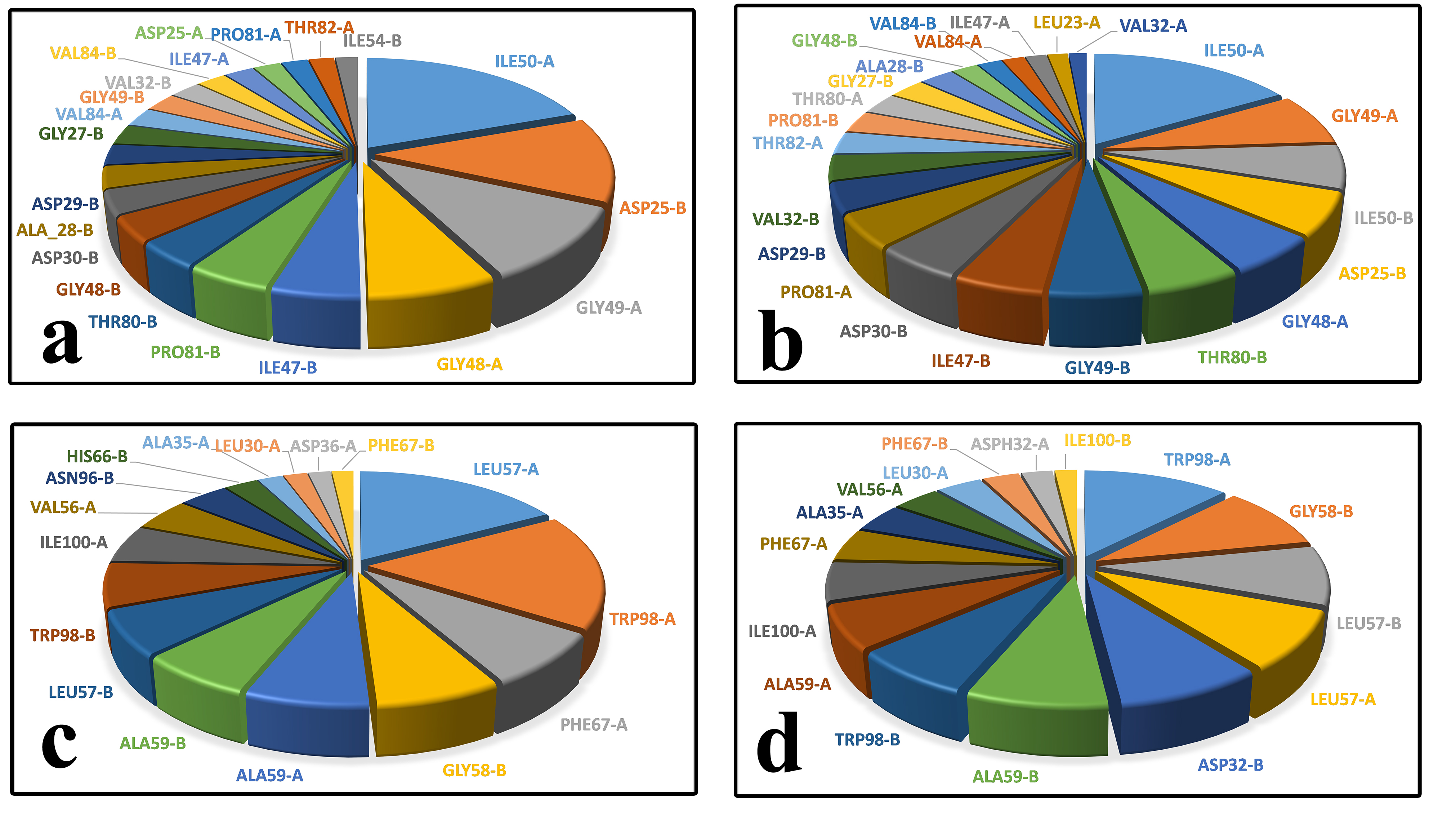

Supplement: S3 Fig — a, b, 1st, and 3rd replica of the Indinavir-HIV protease case. c, b, 1st, and 2nd replica of the Indinavir-HTLV-1 protease case. (TIF) [file pone.0257916.s003.tif]

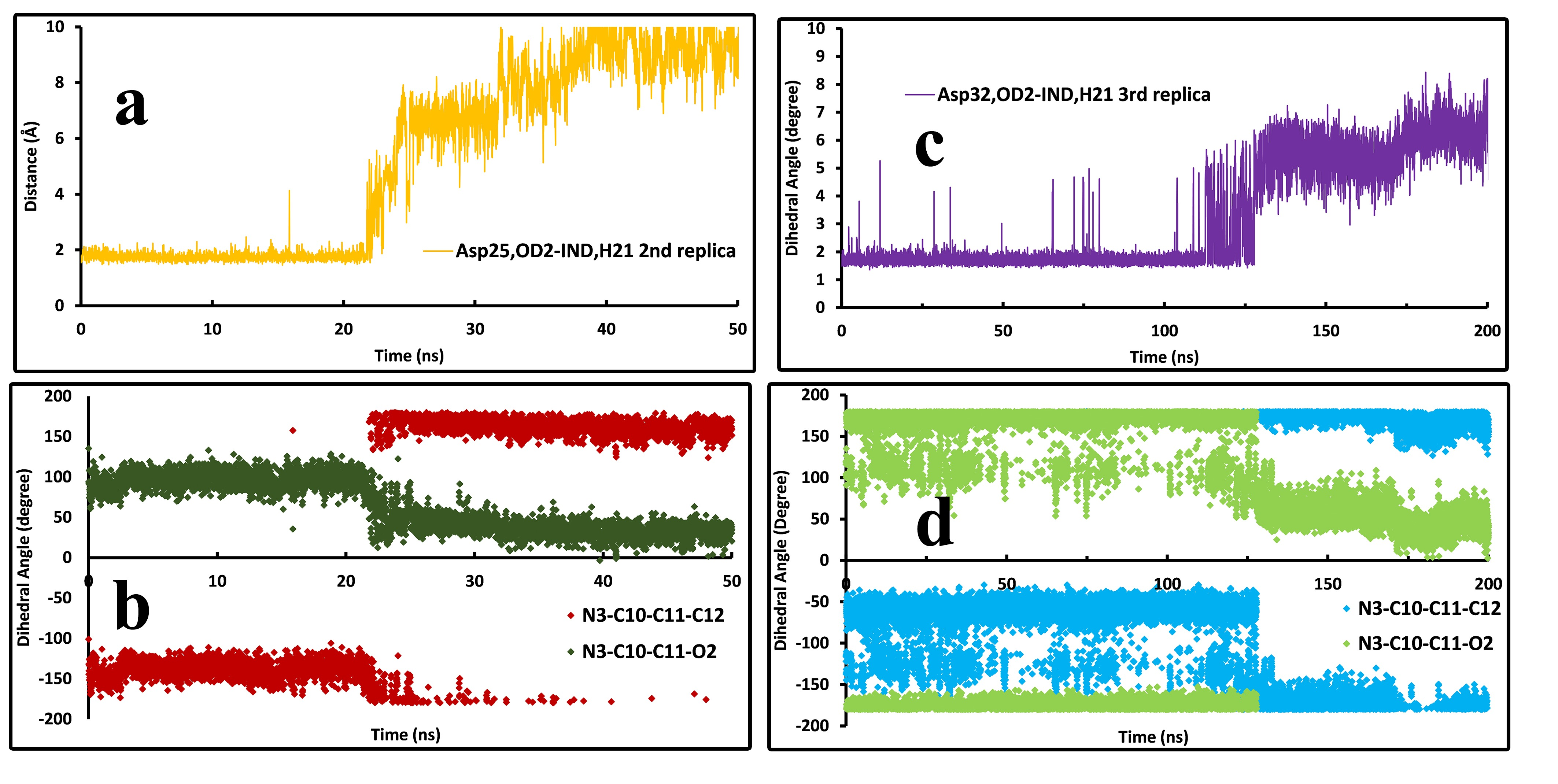

Supplement: S4 Fig — a, The distance between the OD2 atom of Asp25 and the H21 atom of Indinavir in the first 50 ns of the unbinding pathway of Indinavir-HIV complex in the 2nd replica. b, The dihedral angles of the rotatable bond responsible for the rotation of the hydroxyl group of Indinavir in the first 50 ns of the unbinding pathway of the Indinavir-HIV complex in the 2nd replica. c, The distance between the OD2 atom of Asp32 and the H21 atom of Indinavir in the first 200 ns of the unbinding pathway of Indinavir-HTLV-1 complex in the 3rd replica. d, The dihedral angles of the rotatable bond responsible for the rotation of the hydroxyl group of Indinavir in the first 200 ns of the unbinding pathway of Indinavir-HTLV-1 complex in the 3rd replica. (TIF) [file pone.0257916.s004.tif]
