# Supplementary material for: Comparative analysis of the unbinding pathways of antiviral drug Indinavir from HIV and HTLV1 proteases by supervised molecular dynamics simulation
Source: PLoS One. 2021 Sep 27;16(9):e0257916. doi: 10.1371/journal.pone.0257916 (PMC8476009; doi:10.1371/journal.pone.0257916)
Supplement: Attachment — Submitted filename: Response to Reviewers.docx [file pone.0257916.s012.docx]

**Reviewer #2:** The authors have addressed some of my comments. However, overall, I feel that the authors have not done enough improvements to their manuscript so that it can be accepted for publication in PLoS One. Therefore, I would have to recommend rejection. In the following I will some furthermore comments regarding the manuscript.

**Question #1:** I am still not convinced at all with their argument that by performing 50 ps of NVT and NPT equilibration runs after the
frame selection before the 500 ps production runs, they solve the "out-of-equilibration problem" of SuMD. I think this is a point that requires much more consideration and justifications than given in the manuscript.

**Answer #1:** **This study did not focus on the justification of the SuMD method. It focuses on the applications of SuMD in uncovering the unbinding pathways of a ligand. We believe enough explanations have been added to the text.**

**Question #2:** The bottom line is that SuMD can give pathways and mechanisms of ligand unbinding. However, it is clear (see <https://pubs.acs.org/doi/abs/10.1021/acs.jcim.9b01094>) that SuMD cannot give kinetics or dynamics of ligand unbinding due to procedure of running short simulations and selecting a frame for the next iteration. Pathways and mechanisms of ligand unbinding are certainly of interest so insight obtained with SuMD is valuable. However, I would expect user of any method to be honest of the limitations of the method that they are using. In the current version of the manuscript I feel that the authors are not totally honest with the limitations of SuMD.

**Answer #2: Apart from the fact that SuMD is an out-of-equilibrium method, it is a great method to achieve a mechanistic and energetic insight into the unbinding pathway of a ligand, and that was the area we focused on in this study. We did not focus on the kinetics** **parameters of the unbinding pathway. We even mentioned that the duration times of the unbinding pathways of both ligands are in contrast with the real potentials of the ligands: "Although Indinavir inhibits HIV protease much more selectively than the HTLV-1 protease, the overall time needed for the unbinding events of the HTLV-1 protease case was considerably more than that of the HIV protease case. However, many factors govern an inhibitor's activity, and only comparing the overall duration of the unbinding events in three series of replicas is not a correct way of comparing these two cases. This may be due to the inherent limitations of the SuMD method. Because the time window is set to 500 ps, therefore more conformational sampling by ligand is limited."**

**Question #3:** Furthermore, I would like to reiterate my comment regarding talking about talking about Free Energy Landscapes in Figures 3 and 7. A free energy landscape is an equilibrium property, it is the logarithm of the probability distribution in the collective variables considered. This probability distribution would tell us that what is the (relative) population of the different states if we would run an infinitely long MD simulations. To estimate a FEL from a finite MD simulations, one would need to observe many transitions between the different metastable states (e.g., bound and unbound state).
I believe that the authors can agree with me what they present in Figures 3 and 7 are strictly not true equilibrium free energy landscapes. As I said in my previous report, this is still a useful way to present the results from SuMD simulations. It should just be acknowledged that these are not true equilibrium free energy landscapes, but rather a useful way to present the results from the SuMD in a CV space.

**Answer #3: The phrase "out-of-equilibrium" was added to the FEL section to clarify this fact for the readers.**

**Question #4:** Another issue that I noticed while reading <https://pubs.acs.org/doi/abs/10.1021/acs.jcim.9b01094> better (this is the papers from the developers of SuMD where they apply it to ligand unbinding), is that they SuMD procedure used in the current manuscript is different from the one described on page 2 (1805) in <https://pubs.acs.org/doi/abs/10.1021/acs.jcim.9b01094>. The procedure described there is much more intricate while the procedure used in the current manuscripts in much simpler. For example, in [DOI:10.1021/acs.jcim.9b01094] they use the idea of a "productive short MD run" that is not used here. I would have expect the authors to address and acknowledge this in the their manuscript.

**Answer #4: An explanation about the difference was added to the method section.**

**Question #5:** Regarding the data that the authors have made available on Zenodo. The authors state that "Yes - all data are fully available without restriction" and that "all data is available online at: <https://zenodo.org/record/5121095#.YPpq470zbIU>". However, there the authors only include a Gromacs TPR file for each run, and a Gromcas XTC trajectory. Furthermore, there is no information given on how to use the data. In my opinion this does not fulfill the requirements of PLoS of "Authors are required to make all data underlying the findings described fully available, without restriction, and from the time of publication." There is no way of seeing how the conclusion in the manuscript are reached from the data include on Zenodo. I would ask the authors to greatly expand the data included so that the requirement of making available all data needed to reach the conclusions in the manuscript. In my opinion, this should include initial geometries (e.g., Gromcas GRO file), topology files (Gromacs TOP file), and also the script used to perform the simulations. Furthermore, at the very least there should be a readme file describing how to guide people through the data.

**Answer #5: We provided the TPR and the XTC files of each replica in the Zenodo database. These two files are enough for all of the analysis of this study. You can extract the topology, initial geometries, and much more files only by having these two files. Therefore we believe it completely fulfills the journal requirements.**
